# Artificial intelligence in cosmetic dermatology

**DOI:** 10.1111/jocd.16538

**Published:** 2024-08-27

**Authors:** Barbara Kania, Karen Montecinos, David J. Goldberg

**Affiliations:** ^1^ Skin Laser and Surgery Specialists: A Division of Schweiger Dermatology Group Hackensack New Jersey USA; ^2^ Icahn School of Medicine at Mt. Sinai New York New York USA

**Keywords:** artificial intelligence, cosmetic dermatology, machine learning

## Abstract

**Background:**

Cosmetic dermatology is a growing field as more patients are seeking treatments for esthetic concerns. Traditionally, practitioners and patients utilize their own perceptions, current beauty standards, and manual observation to determine their satisfaction with cosmetic interventions. Artificial intelligence (AI) can be introduced into cosmetic dermatology to provide objective data‐driven recommendations to both dermatologists and patients.

**Objective:**

The purpose of this paper is to compose a unified review that illustrates the various facets of artificial intelligence and formulate a hypothesis regarding the new implications of artificial intelligence in cosmetic dermatology specifically.

**Methods:**

A comprehensive search on PubMed was conducted to identify the available information related to AI in cosmetic dermatology. The search was conducted using a combination of keywords including “cosmetic dermatology” and “artificial intelligence.”

**Results:**

The current literature indicates that AI models offer personalized, efficient, and result‐driven outputs that can enhance cosmetic outcomes, patient satisfaction, and overall experience.

**Conclusion:**

Artificial intelligence integration in cosmetic dermatology shows a promising future, offering the ability to analyze vast data sets and deliver a tailored patient experience. By incorporating AI into cosmetic dermatology, there is an opportunity to balance evidence‐based decision‐making with the artistic human touch of cosmetic dermatologists.

## 1 INTRODUCTION

Cosmetic dermatology is a continuously evolving field as per the demands of an aesthetic‐conscious society. This specialized branch of dermatology focuses on enhancing the appearance of the skin, targeting concerns such as wrinkles, photodamage, skin laxity, and pigmentation. Cosmetic dermatologists are required to approach these concerns with a blend of evidence‐based scientific understanding and artistic techniques to help patients achieve their desired cosmetic outcomes. As an artistic discipline, cosmetic dermatology is unavoidably prone to subjectiveness when measuring success in esthetic outcomes. Traditionally, the success of a cosmetic intervention is gauged by individual perception, current beauty standards, and manual observation; all of which are highly subjective measures that are susceptible to biases. In the light of these challenges, there is a growing interest in the potential role of artificial intelligence (AI) in cosmetic dermatology.

## 2 METHODS

A comprehensive search on PubMed was conducted to gather all the published material related to the current implications of AI in medicine, specifically focusing on AI in the field of dermatology. Fifty‐three scholarly articles were analyzed using keywords such as “cosmetic dermatology” and “artificial intelligence.” Thirty‐three of these articles were selected that provided definitions for our key terms to compose a unified review that illustrates the various facets of AI.

### 2.1 Artificial intelligence in cosmetic dermatology

Artificial intelligence is the programming of machines, computers, or software to simulate human intelligence in order to perform cognitive functions such as learning, decision‐making, and problem‐solving.^1^ This can be accomplished via the utilization of artificial neural networks (ANN), machine learning (ML), deep learning (DL), and convolutional neural networks (CNN).^2^ The application of AI in medicine is still fairly new, and while there has not been an exact number established of healthcare providers who are currently using AI in practice, the American Medical Association (AMA) conducted a survey in August 2023 to investigate its usage in clinical practice.^3^ Surveying 1081 physicians, the AMA reported that AI tools were in use by 38% of physicians to assist them in accomplishing the following tasks: creation of discharge instructions, care plans, or progress notes (14%), documentation of billing codes, medical charts or visit notes (13%), translation services (11%), and assistive diagnosis (11%).^3^

In the field of dermatology, AI has been used to diagnose and classify melanoma images at a more accurate rate than dermatologists.^4^ The growing demand for cosmetic treatments now expands AI's role beyond medical dermatology. Cosmetic dermatology can be defined as the focus on enhancing the physical appearance of the skin by improving overall quality, addressing the signs of aging, and other esthetic concerns.^5^, ^6^ While patients vocalize their concerns, treatment decisions are made using subjective parameters based on the dermatologist's clinical experience and expertise.^7^

Integrating AI into cosmetic dermatology, it can offer a more personalized objective approach to cosmetic procedures. Prior to treatment with neuromodulators or fillers, DL within computer vision technology has been used to identify fine lines in patients to identify the best mode of treatment.^8^ As some dermatological conditions may present similarly, AI can analyze images from a database to reduce human error.^9^ The integration of AI into cosmetic dermatology will enable dermatologists to make data‐driven decisions, predict treatment outcomes, and customize treatment plans to better meet the needs and expectations of the patient.

### 2.2 Artificial intelligence in cosmetic consultations and assessing outcomes

Cosmetic consultations are arguably one of the most time‐consuming tasks in cosmetic dermatology. Each patient presents with different concerns, skin types, and predispositions that demand an attentiveness from their dermatologist to provide the appropriate treatments with the highest likelihood of reaching their aesthetic outcomes. During the consultation, patients are able to discuss their cosmetic concerns and goals but must rely on the dermatologist's opinion to recommend the best course of action.^10^ AI image analysis can be utilized to save time during consultations by quickly diagnosing cosmetic conditions.^11^

Skin quality classifications are critical elements of cosmetic consultations. While traditional assessments of skin quality rely on manual observation, which can be highly subjective and challenging to quantify, the utilization of image recognition and AI analysis can assist practitioners in providing enhanced accuracy and efficiency.^12^ Chirikhina et al. used Epsilon values, contact capacitive imaging, and high‐resolution ultrasound imaging to noninvasively estimate the water content of the skin.^13^ They measured water content in multiple facial regions, including the cheek, chin, corner of the eyes, forehead, lips, neck, and nose.^13^ Several DL algorithms were evaluated for the ability to classify contact capacitive images, with DenseNet201 (83.8%) showing the highest accuracy to actual water content in the subject's skin.^13^ Zegour et al. also measured skin hydration levels using high‐resolution magnetic resonance (MRI) imaging.^14^ Using DenseNet and U‐net algorithms to compare an algorithmic output and a variant of human‐derived segment, these researchers showed that ML techniques yielded a skin hydration assessment that was notably closer to manual assessment by expert cosmetic dermatologists.^14^ This offers a noninvasive method to measure skin hydration that is comparable to human measurement. Assisting practitioners with saving time and offering an objective parameter when evaluating success after cosmetic treatments, or as a baseline measurement of water content in a patient's skin at consultation.

AI can also transform the process of assessing skin barrier health in patients during their cosmetic consultation. Traditional methods rely on measuring transepidermal water loss (TEWL), which is the amount of water that evaporates from the skin.^15^ Healthy skin barriers have the capacity to retain water, so TEWL measurements can allow practitioners to gauge intact skin versus skin barrier dysfunction.^16^ Despite its importance, traditional measurements can be very time‐consuming and tedious for practitioners. ML algorithms can be employed to analyze skin images and predict skin barrier function in a quick, efficient, and accurate manner. Koseki et al. utilized ML and topological data analysis, which enables the identification of structural features from a skin image taken by a microscope, to predict TEWL all through the use of AI.^15^ The systems trained in this study achieved good accuracy and allow for a more systematic way of assessing skin barrier health that is otherwise not as easily accessible in current‐day assessments.^15^

Sebum assessment is essential for patients seeking cosmetic treatments that target conditions such as acne. While traditional assessment of sebum production relies on subjective manual observation, AI has the ability to replace this process with a more measurable objective parameter. Borade et al. conducted a study that examined different AI systems including SVM, VGG‐16, and ResNets in their ability to classify preprocessed skin images.^17^ Their findings displayed that ResNets resulted in a 98% accuracy which surpassed other model performances.^17^ Similarly, Kothari et al. trained a CNN to estimate the oiliness level of each image taken of patients’ faces and classify them based on their respective types (i.e., normal, dry, oily, combination).^18^ Utilizing facial detection techniques, this system was able to assess and categorize them into their respective distinctions with high accuracy.^18^

Many patients seek cosmetic procedures, including rejuvenating lasers and fillers, to regain the look of fuller, thicker skin. Skin thickness is an important quality to assess when considering the right treatment plan for patients, and can provide a better understanding for practitioners regarding their patient's areas of concern. Unfortunately, traditional assessment of skin thickness involves biopsying the skin and utilizing clinical correlation, which can be time‐consuming and invasive.^19^ AI has significantly optimized this process as seen in Hu et al.'s study in which the researchers utilized DL and image processing to measure epidermal thickness to determine collagen production or loss of collagen without biopsying the patient.^20^ This noninvasive approach can provide practitioners with an easily accessible way to measure skin thickness at baseline, and then afterward to show improvement following cosmetic interventions.

AI can help in identifying skin lesions and characteristics that patients often mention during consultation. The identification of specific colors and pigments on patient's faces can play a crucial role in determining the appropriate laser type and wavelength to be used for their treatment. Holcomb studied Canfield's VISIA AI skin system which utilizes cross‐polarization and UV illumination mechanisms to measure both surface‐level and subsurface‐level skin conditions.^21^ Utilization of this technology in clinical practice offers fine‐tuned detection of distinct color characteristics that can lead to brown and red components of color concentration in conditions such as spider veins, port wine stains and hyperpigmentation that can be then monitored more objectively and visualized more efficiently for the dermatologist to select the best treatment plan for their patient.^21^ Yang et al. classified 12 816 photos of benign and pigmented facial skin lesions at the Hospital for Skin Diseases at the Chinese Academy of Medical Sciences from 2004 to 2016 based on skin condition.^11^ These skin conditions included acquired nevi of Ota, melasma, cafe au lait spots, freckles, seborrheic keratosis, and nevi of Ota. Utilizing AI, the system resulted in comparably accurate results to three expert dermatologist assessments.^11^ The ability to categorize skin images holds the potential for assisting in initial self‐diagnosis and could integrate into a decision support system for dermatologists, thereby saving time on cosmetic consultations. Similarly, Flament et al. assessed the severity of various skin conditions using 1041 self‐portrait images.^22^ Findings indicated a robust correlation between automated and dermatologist results for several conditions including forehead wrinkles, periorbital wrinkles, nasolabial folds, and diffuse redness.^22^ The advances in image recognition and analysis technologies not only facilitate aesthetic assessments but contribute significantly to the development of cosmetic treatment plans at the time of consultation.

### 2.3 Artificial intelligence in treatment prediction and progress

Artificial intelligence offers great strides in predictive modeling to assist providers with closing the “gap” between the patient and the unknown outcome of their treatment plans. For vitiligo patients, ANN models have been used to predict the number of Excimer laser treatments needed and this application of predictive modeling could be utilized for cosmetic laser treatments to treat dyschromia or acne scarring.^8^ Given the potentially lengthy and costly nature of cosmetic treatments, AI can recommend treatments and use predictive modeling to empower the patient to select the correct treatment for them, minimizing the need for retreatments to accomplish their esthetic goals. Furthermore, AI can predict the treatment efficacy of targeted cosmetic treatments.^23^

Supporting this, Shah et al. demonstrated the ability to generate a precise 3D facial image following a simulated rejuvenation process.^24^ The model was used as an input for generating 3D facial scans, which were then used to identify facial landmarks for filler injection sites.^24^ This study introduced a model that predicted the quantity of dermal filler required for facial application, utilizing a multilayered neural network architecture.^24^ This approach yielded an accuracy of 62.5% to dermatologist evaluation, surpassing other methods at the time.^24^ Artificial intelligence‐simulated postoperative images derived from baseline images serve as a tool for patients to make informed decisions regarding their treatment options. The predicted results also provide an estimate of the probable costs, setting expectations for what patients are investing their money in. This can allow for better planning for both the practitioner and patient when deciding which treatments to prioritize, and which to revisit if needed.

Artificial intelligence can be very helpful in its ability to track and measure treatment progression accurately in real time. Traditionally, the progress of cosmetic treatments is monitored through subjective analysis as the provider is carrying out the procedure. Providers rely on their experience and knowledge to look for clinical endpoints (i.e., frosting in chemical peels, pinpoint bleeding in microneedling) to determine whether the patient is tolerating the procedure well. However, this approach may not reflect the accuracy of the treatment's true progression in real time. Enter AI, which offers a more objective measure for tracking treatment progression. For example, Erdogan et al. used a DL algorithm and KEBOT for postoperative evaluation of the Follicular Unit Extraction (FUE) hair transplantation procedure.^25^ Through AI, they were able to conduct an analysis with object detection followed by hair thickness estimation.^25^ The surgeon was then presented with the postoperative prediction, which was instrumental in strategizing the surgical procedure. Through sophisticated algorithms and image analysis, AI can accurately measure the health of hair follicles during hair transplantation, providing the surgeon with a likelihood score that indicates the probability of a hair follicle surviving the transplant.^25^ Artificial intellgence's ability to quantify improvement throughout a patient's series of cosmetic treatments offers a clearer picture of the treatment's effectiveness and a more accurate understanding of treatment progression.

### 2.4 Artificial intelligence in patient education

Artificial intelligence can be utilized as an interactive education tool for patients to maintain their cosmetic outcomes following their treatment. Shi et al. created a “skincare mirror,” a system that predicts a person's appearance after using specific skincare products.^26^ This interactive tool provides users with a personalized visualization of potential outcomes, enabling them to make more informed decisions regarding their skincare regimen.^26^ The study revealed varying behavioral patterns among users. Those who utilized the skincare mirror more often tended to explore a greater number of products, while spending comparatively less time reading through the product descriptions.^26^ Interestingly, the male cohort, who generally lacked skincare knowledge, exhibited higher levels of satisfaction with the system compared to other groups.^26^ This suggests that AI‐based education tools can play a pivotal role in enhancing user engagement and satisfaction, particularly among demographics that are less familiar with skincare Table 1.

**TABLE 1:** Tasks required from cosmetic dermatologists and the corresponding traditional approach, newly AI‐guided approach, and the outcomes of AI intervention of these tasks.

Task	Traditional practice	AI practice	Potential outcomes of AI	References
Candidate selection	Provider determination based on prior experience and manual observation of patient's concerns	DL algorithms identify facial features (i.e., rhytids) using image detection	More accurate candidate selection Less time spent manually identifying areas of concern	8, 11
Skin quality classification	Manual observation	Image recognition using DL algorithm to predict skin hydration	Provides a parameter to measure skin quality before and after treatment to assess the success of treatment objectively	13, 14
Skin barrier health assessment	Measuring TEWL (time‐consuming, tedious)	ML algorithms to analyze images of skin and predict TEWL	Faster, more accessible, and accurate measurement of TEWL	^15^
Sebum production assessment	Manual observation	Facial detection and comparison to database of other patients to categorize patient's sebum level more objectively	Quicker categorization of a highly subjective finding	17, 18
Skin thickness assessment	Skin biopsy (time‐consuming, invasive)	DL and image processing to measure epidermal thickness and quantify collagen levels	Noninvasive mechanism to obtain skin thickness measurement which can be used to determine efficacy of cosmetic interventions	^20^
Color characterization and detection	Manual observation	Canfield's RBX® technology utilizes cross‐polarization and UV illumination to measure red and brown skin components of color concentration	Finer visualization of subsurface melanin and vascular conditions such as spider veins and hyperpigmentation	^21^
Diagnosing skin conditions and lesions	Manual observation and diagnosis based on the knowledge base of the provider	Image recognition and comparison to database to categorize skin images based on diagnosis	Highly accurate, automated diagnosis of skin lesions that saves the provider time	11, 22
Treatment recommendations	Manual diagnosis of skin condition based on provider knowledge base to then recommend treatments shown to improve aesthetic concern	Image recognition to diagnose skin condition and provide treatment recommendation based on compiled data from AI algorithms with corresponding success rates to provide treatment recommendations for provider	Faster, efficient and accurate selection of treatment recommendations from AI for the provider to reference	27, 28
Predicting outcomes	Setting expectations with patients based on provider's experience with treatment outcomes	AI generation of simulated 3D images specific to patient's face based on database analysis of all available patient outcomes to create a relatable and personalized “posttreatment” photo	Tangible results using predictive modeling that allows patients to make more informed decisions on treatments	^24^
Financial investment assessment	Provider recommendation of specific number of treatments to set financial investment expectation based on prior experiences	AI generation of simulated 3D images specific to patient's face based on database analysis of all available patient outcomes to serially trace out predicted outcomes after inputted number of treatments	Better transparency around financial investment expectation for both patient and provider	^24^
Treatment progression evaluation in real time	Manual observation of clinical endpoints (i.e., frosting, pinpoint bleeding)	DL algorithm and object detection analysis to measure specific parameters (i.e., hair follicle thickness) to provide success rate statistics (i.e., hair transplant success rate)	Provides real‐time reflection of treatment progress and AI‐estimated success rates, allowing provider to change treatment parameters to maximize success	^25^
Patient education	Discharge paperwork, lengthy discussions with patients to educate them on product recommendations based on provider preference	Predictive AI algorithms that use patient's photos and create personalized visualization of outcomes on specific products based on their phenotype and data on ingredients in each product	AI‐based tools can enhance user compliance and satisfaction with at‐home skincare regimens	27, 28
Evaluation of cosmetic treatment outcomes	Subjective before and after photograph analysis	Comparing photos and several measurable parameters with database of images to compare outcomes objectively with other patients who underwent similar treatments	More objective parameters that can be used to measure outcomes of treatment that are otherwise tedious or inaccessible to measure currently	11, 22

Abbreviations: AI, artificial intelligence; DL, deep learning; ML, machine learning; TEWL, transepidermal water loss.

Moreover, AI‐driven treatment recommendations offer a more personalized and realistic outcome for patients. Huang et al. developed Alluring, a cloud‐based system for dermatological analysis of the skin and scalp.^27^ This system uses skin images and undergoes comprehensive analysis for factors like moisture, oil, sensitivity, color, pore size, and distribution.^27^ The state‐of‐the‐art object detection system YOLO‐v2 is used to process these factors and suggest a tailored product recommendation that is specific to the patient.^27^ Similarly, Liu et al. developed a method for recommending cosmetic products by integrating genetic data related to consumer skin health, product data, and demand factors based on phenotype.^28^ They also considered data on the relationship between ingredients and their functions. The utilization of genetic data and consideration of trade‐offs between phenotypic demands led to an improvement in the recommendation performance that ultimately resulted in a better cosmetic outcome for patients.^28^

### 2.5 Challenges and future directions

With the benefits that AI can bring, there are also limitations. Some of these issues include data quality.^29^, ^30^ Artificial intelligence relies heavily on the data sets that are used when programming, so if there are any errors present, this will affect the outputs. Sufficient data must be inputted to achieve high accuracy and representation.^9^ The absence of adequate data sets and data imbalance can inhibit AI from effectively learning and producing reliable results.^9^

Artificial intelligence has inherited many negative characteristics from the human mind such as unjustified biases and discrimination. In 2016, a beauty contest was judged by AI and resulted in almost all the winners being women with fair skin.^31^ If the data sets used to develop the AI are not diverse and representative, such as being trained on a single skin type or an undiversified socio‐demographic population, the AI might disproportionately favor specific traits or features.^9^ Ethnic considerations, such as the way different ethnic groups age or their standards of beauty, should be accounted for to avoid discrimination in AI algorithms.^32^ Future research should focus on developing strategies to mitigate these biases, ensuring equitable and fair representation of all skin types in AI algorithms.

There has been growing concern with the ethical and regulatory aspects of AI adoption particularly in ensuring patient privacy and data security.^33^ Machine learning relies on clinical imaging data, but it is challenging to obtain consent as the images are identifiable which does not ensure the patient's privacy.^34^ Furthermore, the field of dermatology lacks standardized practices for image capturing and storing, making it even more difficult for patients and practitioners to adopt the use of AI.^34^ Measures need to be taken to protect user confidentiality and ensure data security as AI continues to be integrated into health care. The incorporation of AI into medical school curriculums could ease the transition into a more tech‐driven healthcare environment. By equipping future healthcare professionals with knowledge about AI, they will be better prepared to leverage this technology to enhance patient care.^35^

## 3 CONCLUSION

Artificial intelligence integration in cosmetic dermatology shows a promising future, offering the ability to analyze vast datasets and deliver a tailored patient experience. By incorporating AI into cosmetic dermatology, there is an opportunity to balance evidence‐based decision‐making with the artistic human touch of cosmetic dermatologists. It is important to emphasize the importance of the word “balance,” as AI will be a complimentary tool rather than a replacement for the essential human expertise that the field of cosmetic dermatology demands. This integration can revolutionize cosmetic consultations, treatment planning, progress analysis, and patient education and ultimately lead to further major advancements in cosmetic dermatology.

## ETHICS STATEMENT

The authors declare that human ethics approval was not needed for this study.

## Data Availability

The data that support the findings of this study are available from the corresponding author upon reasonable request.
